# Lung cancer burden attributable to ambient particulate matter: a nationally representative population-based case-control study

**DOI:** 10.1038/s41416-025-03207-x

**Published:** 2025-10-06

**Authors:** Rawan A. N. Alhattab, Jennifer M. McKinley, Ruth F. Hunter, Claire M. Delargy, Sara M. Wallace, Damien Bennett, Deirdre Fitzpatrick, Helen Mitchell, Bernadette McGuinness, Angela Scott, Gareth McKay, Liacine Bouaoun, Valerie McCormack, Daniel R. S. Middleton

**Affiliations:** 1 Centre for Public Health, Queen’s University Belfast, Belfast, UK; 2 Ibn Sina University for Medical Sciences, Amman, Jordan; 3 Geography, School of Natural and Built Environment, Queen’s University Belfast, Belfast, UK; 4 Northern Ireland Cancer Registry, Centre for Public Health, Queen’s University Belfast, Belfast, UK; 5 Environment and Lifestyle Epidemiology Branch, International Agency for Research on Cancer (IARC-WHO), Lyon, France

**Keywords:** Lung cancer, Epidemiology

## Abstract

**Background:**

Particulate matter with a diameter of 2.5 micrometers or less (PM_2.5_) is a known lung carcinogen, but its impact in low-pollution settings is less understood. We assessed the association between long-term PM_2.5_ exposure and lung cancer risk in Northern Ireland (NI), a region with relatively low air pollution levels.

**Methods:**

We conducted a population-based case-control study using data from the Northern Ireland Cancer Registry and the Northern Ireland Cohort for the Longitudinal Study of Ageing. The study included 917 lung cancer cases diagnosed in 2014 and 8,088 controls without lung cancer. Eight-year average PM_2.5_ exposure was estimated by linking residential postcodes to 1 km² resolution pollution maps. Fully adjusted logistic regression models were used, controlling for key confounders including smoking status and deprivation index to estimate odds ratios (ORs) and their 95% confidence intervals (95% CI), and population attributable fractions (PAFs).

**Results:**

Individuals in the highest PM_2.5_ tertile (>9.6 µg/m³) had a 37% increased lung cancer risk (OR: 1.37; 95% CI: 1.12–1.68) compared to the lowest tertile (<7.4 µg/m³). The association was stronger in women (OR: 1.79; 95% CI: 1.32–2.44) and not detected in men. Exposure above 10 µg/m³ accounted for 10% of cases, approximately 137 preventable lung cancers annually.

**Discussion:**

Even in low-pollution regions, PM_2.5_ contributes to lung cancer risk, especially in women. Strengthened air quality measures are needed to reduce preventable disease.

## Background

Lung cancer remains the leading cause of cancer incidence and mortality worldwide – with almost 2.5 million new cases and over 1.8 million lung cancer deaths estimated globally in 2022 [1]. In the UK and Northern Ireland (NI), 50,700, and 1367 new cases respectively are diagnosed annually [2, 3]. Among cases diagnosed in NI, 44.4% are diagnosed with advanced Stage IV disease, consistent with other European settings [3], beyond which curative treatment is unfeasible and only 1.6% of patients survive beyond 5 years [3], highlighting the dire prognosis of this cancer and the importance of primary and secondary prevention strategies.

Tobacco smoking is the largest contributor to the lung cancer burden in high-income countries, estimated to account for 68.7% of cases in NI, lower than the UK-wide estimate of 72.2% [4]. Nevertheless, a decline in smoking prevalence means that the proportion of lung cancer cases who are lifelong never smokers is rising [5], making the investigation of other risk factors important. There is debate among the scientific community regarding the next most substantial contributor to lung cancer burden. In NI, occupation (13.2%), air pollution (5.6%), and ionising radiation (i.e., radon gas) (4.2%) are the next largest estimated fractions [4], though based on generalized assumptions.

Air pollution poses a public health threat that almost all populations are exposed to. The World Health Organization (WHO) report that 99% of people breathe air that exceeds their recommended Air Quality Guideline (AQG) annual average of 5 µg/m^3^ [6]. As well as increasing the risk of numerous respiratory and cardiovascular outcomes [7], outdoor air pollution has been evaluated as Group 1 (‘carcinogenic to humans’) by the International Agency for Research on Cancer (IARC) [8]. Rates of adenocarcinoma, a histological subtype of non-small cell lung cancer (NSCLC) accounting for the majority of cases worldwide, and 27–40% in NI [3], appear to be particularly driven by air pollution. Modelling the estimated 2022 worldwide lung cancer burden by subtype found that 27% of adenocarcinomas—194,864 annual cases—are attributable to ambient particulate matter [5].

Particulate matter, often a proxy for air pollution broadly, was evaluated separately by IARC and classified as Group 1 [8]. Particulate matter with a diameter of 2.5 µm or less (PM_2.5_) constitutes a mixture of pollutants that penetrate deep into the alveoli and are absorbed into the bloodstream [9]. It has been proposed that PM_2.5_ acts as a tumour promotor following exposure of lung cells harbouring oncogenic driver mutations which are particularly common in the tumours of non-smokers [10]. Numerous studies of cancer in humans have found positive associations between PM_2.5_ exposure and lung cancer even at exposure levels below the WHO AQG [11].

While the UK ranks 112th in global standings for ambient PM_2.5_, measurements recorded in UK cities regularly exceed the former WHO AQG [12] of 10 µg/m^3^ [13]. The major sources of PM_2.5_ in NI are industrial combustion, emissions from vehicle exhausts, and domestic fuel combustion. Incidentally, Belfast is the third most car-dependent city in Europe [14]. Despite known health risks of ambient air pollution, only a few lung cancer observational studies have been conducted in the UK [10, 15] and none in NI. Moreover, studies elsewhere have successfully employed geospatial air pollution maps [10, 15], the extent of which covers NI at 1 km^2^ resolution, where they have yet to be used to investigate lung cancer. The availability of these metrics and of population-based cancer statistics collected by the NI Cancer Registry (NICR) [16] present an opportunity to investigate the air pollution attributable lung cancer burden.

In this population-based, nationally representative case-control study, we aimed to investigate ambient PM_2.5_ concentrations and lung cancer risk and estimate the population attributable fraction of lung cancer in NI due to PM_2.5_.

## Methods

### Ethical approval

NICR has ethical approval from the Office for Research Ethics Committees (ORECNI) (Ref: 20/NI/0132, IRAS project ID: 288121), for the collection and use of routinely collected data, relating to cancer patients, within the fields of health and social care research. Ethical approval for the NICOLA study was obtained from the QUB School of Medicine, Dentistry and Biomedical Sciences Research Ethics Committee (Reference: 12/23).

### Study design, setting, and data

We constructed a population-based case-control study in NI, a country with a population of 1.9 million [17]. The study used two preexisting data sources of separate origin. The NI Cancer Registry (NICR) is a population-based registry collecting data on all cancer patients diagnosed in NI since 1993 [16]. The NI Cohort for the Longitudinal Study of Ageing (NICOLA) includes a nationally representative sample of ~8,500 adults living in private residences over age 50 years recruited across two waves (1: 2013-2016; 2: 2017–2019). Given that few lung cancer occur below age 50, NICOLA participants are proposed as an appropriate control group representative of the population from which NI’s cancer cases arise.

### Case definition and ascertainment

Primary lung cancer cases (ICD-10 C34) diagnosed in 2014 and living in NI at time of diagnosis were selected for this analysis as smoking status data (a leading cause of lung cancer and potential confounder) collected during a routine audit [18] were available for these patients. A flow diagram detailing exclusion criteria of cases and controls is presented in Fig. S1. Of 1219 cases, 263 (22%) were excluded due to missing smoking status. This missingness was uniformly distributed across cases due to differences in completeness of electronic databases during the audit. Comparison to the smoking status distributions of previous audits generated comparable smoking prevalences to that of current cases. Thirty-six cases diagnosed below age 50 were excluded to reflect the age distribution of the comparative NICOLA controls. Three cases were excluded as their postcode coordinates could not be located, yielding 917 cases with both smoking and PM_2.5_ data.

### Control group

The control group comprised NICOLA Wave 1 participants recruited between 2013 and 2015. These years are one year either side of the cases’ diagnosis year, i.e. similar to the recruitment schedule of a typical case-control study. As shown in supplementary information Fig. S1, 23 participants with either a historical or incident cancer diagnosis were excluded from the control group through linkage to the NICR, as well as 88 with missing smoking data and 3 with a missing date of birth, 197 household spouses aged below 50 years old, and 33 for whom PM_2.5_ exposure could not be determined. This resulted in 8088 controls.

As the ideal control group consists of a random sample of the population from which cases arose, the representativeness of NICOLA participants with regards to primary exposure of interest was assessed by comparing their 8-year annual average PM_2.5_ distribution to that of all NI residents using a 2011 census by postcode headcount linked to PM_2.5_ as described below. No bias in PM_2.5_ exposure prevalence was observed (Table S1), supporting the representativeness of controls.

### PM_2.5_ exposure characterisation

**Fig. 1:**
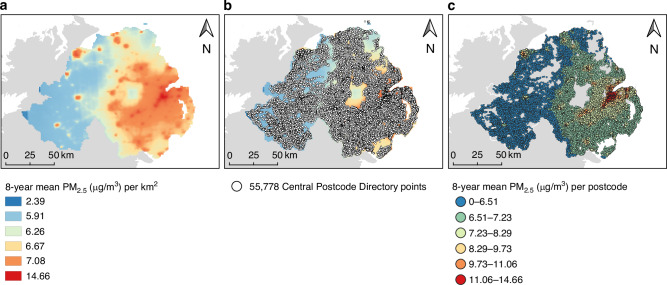
Air pollution exposure characterisation among study participants. Map (**a**) displays the 8-year mean PM_2.5_ exposure across Northern Ireland (NI) in a raster layer at 1 km^2^ resolution. Map (**b**) shows the distribution of postcodes from the Central Postcode Database (CPD) across Northern Ireland. Map (**c**) illustrates the estimated PM_2.5_ values at each postcode in the CPD. Postcodes of study participants were then extracted from this air pollution-linked CPD dataset.

Annual csv files of mean ambient PM_2.5_ concentrations at 1 km grid squares covering NI were downloaded from the Department of Environment Food and Rural Affairs (DEFRA) for 2006 to 2013 [19]. These data are generated by modelling the concentrations measured by certified gravimetric analysis at 23 monitoring stations around the region [19, 20]. Files were imported into a Geographic Information System (GIS) using the Quantum GIS (QGIS) software (version 3.36.1) and converted into a shapefile which was then rasterized for point sampling. Postcode coordinates for the 55,778 alphanumeric postcodes registered in the NI Central Postcode Directory (CPD) [21] were imported into QGIS and used to extract PM_2.5_ values at underlying raster cells using the ‘sample raster values’ tool. This process is demonstrated in Fig. 1. Postcodes provided by participants representing their residence at either diagnosis (cases) or interview (controls) were joined to this CPD dataset of postcode-specific PM_2.5_ estimates. Mean PM_2.5_ concentration during 2006–2013 was calculated for participants, representing the mean ambient PM_2.5_ at participants’ postcodes in the 8 years preceding the earliest interview/diagnosis date of the study participants. Annual means of all eight individual years were highly correlated.

### Covariates

For cases, age, sex and residential location at diagnosis are routinely collected as per NICR’s registration process. Smoking status (current, ex-, never smoker) was obtained as part of NICR’s audit process by interrogating medical records in linked databases. For controls, all variables were collected during personal interviews. Variables were recoded/relabelled into a harmonized format between cases and controls. The NI Multiple Deprivation Measure 2010 [22] was applied at the postcode level. The measure comprises seven domains of deprivation, primarily including income and employment, but also including health and disability, education, skills and training, proximity to services, living environment, and crime and disorder [22].

### Statistical analysis

Primary exposure of interest was continuous (8-year annual mean—µg/m^3^) and categorical PM_2.5_. For the latter, tertiles of PM_2.5_ were determined in controls (high: >9.62 µg/m^3^; medium: 7.42–9.62 µg/m^3^; low: <7.42 µg/m^3^). χ2 tests were used to investigate associations of possible confounders with PM_2.5_ tertiles in controls. Odds ratios (ORs) and 95% confidence intervals (CIs) of lung cancer associated with PM_2.5_ exposure tertiles were calculated using logistic regression models. In *Model 1*, crude ORs were generated. *Model 2* had minimal adjustment for age (5-year categories) and sex. *Model 3* was fully adjusted with terms for smoking (current, ex-, never smoker) and deprivation quintile (1—least deprived; 5—most deprived). Subgroup analyses were investigated in men, women, under 65 years old, over 65 years old, ever-smokers, and never-smokers. The effect was also investigated in geographic areas excluding Belfast, as the city has a shipbuilding history and rates of mesothelioma are known to cluster here due to asbestos—a confirmed lung carcinogen [23]. In addition, we examined associations with lung cancer separately by histological subtype: adenocarcinomas, squamous cell carcinomas, all non-small cell carcinomas, and small cell carcinomas. To graphically display the dose-response relationship between lung cancer risk and continuous PM_2.5_ exposure, PM_2.5_ was also included as a natural cubic spline with five degrees of freedom.

Using fully adjusted ORs, assuming causality, we used Miettinen’s formula [24] (Eq.( 1)) to estimate the population attributable fraction (PAF, %) of lung cancer attributable to PM_2.5_ exposure above the former WHO AQG of 10 µg/m^3^; i.e. the proportion of avoidable lung cancer s if annual average ambient pollution at their postcodes was below this level.

1 $${{{\rm{PAF}}}}={{{\rm{pc}}}}\times ({{{{\rm{RR}}}}}_{{{{\rm{adj}}}}}-1)/{{{\rm{RR}}}}{{{\rm{adj}}}}$$

where PAF is the population attributable fraction, pc is the proportion of cases exposed to >10 µg/m^3^ 8-year annual PM_2.5_, and RR_adj_ is the fully adjusted relative risk (*Model 3* OR for binary exposure). Calculations were reported based on the point estimate, and upper and lower 95% CIs.

As a precautionary statistical modelling approach, an alternative matched design was constructed whereby each case was individually matched with up to four randomly selected controls of the same sex within ±1 year of age. *Models 1–3* were then repeated using conditional logistic regression. All statistical analyses were generated using the R programming language (version 4.2.2.) [25].

## Results

### Participants and exposure characteristics

Characteristics of lung cancer cases (*n* = 917) and controls (*n* = 8,088) are presented in Table 1. Controls were a mean of 5.8 years younger than cases and included a higher proportion of females (54.7%) compared to cases (46.1%). Cases included a higher proportion of current/ex-smokers (94.7%) than controls (51.6%). A higher proportion of cases’ postcodes belonged to the most deprived areas (29.6% in quintile 5) compared to controls (14.7%), as well as being located at higher 8-year mean PM_2.5_ concentrations– 9.4 µg/m^3^ versus 8.8 µg/m^3^ for controls. The most common lung cancer subtype reported was adenocarcinoma (32.1%), followed by squamous cell carcinoma (24.9%) and small cell carcinoma (12%). Potentially confounding variables were tabulated against PM_2.5_ tertiles in Table S2 with χ² test *p*-values. Significant associations were observed for age (*p* = 0.02), smoking (*p* < 0.001), and deprivation (*p* < 0.001).

**Table 1:** Demographic and exposure characteristics of lung cancer cases and NICOLA controls.

Characteristics	Cases *n* = (column %)	Controls *n* = (column %)
Total	917	8088
**Age (years) at diagnosis/interview**		
Mean (SD)	71.1 (8.9)	65.3 (10.0)
**Age group**		
50–54	32 (3.5)	1300 (16.1)
55–59	67 (7.3)	1446 (17.9)
60–64	122 (13.3)	1362 (16.8)
65–69	143 (15.6)	1372 (17.0)
70–74	201 (21.9)	1062 (13.1)
75–79	192 (20.9)	729 (9.0)
80–84	105 (11.5)	488 (.0)
>= 85	55 (5.9)	329 (4.12)
**Sex**		
Male	494 (53.9)	3662 (45.3)
Female	423 (46.1)	4426 (54.7)
**Smoking status**		
Current	439 (47.9)	1350 (16.7)
Ex-smoker	429 (46.8)	2821 (34.9)
Never	49 (5.3)	3917 (48.4)
**Deprivation class**		
Quintile 1 (Least deprived)	111 (12.1)	1773 (21.9)
Quintile 2	171 (18.6)	1749 (21.6)
Quintile 3	165 (18.0)	1806 (22.3)
Quintile 4	199 (21.7)	1569 (19.4)
Quintile 5 (Most deprived)	271 (29.6)	1191 (14.7)
**PM**_**2.5**_ **exposure**		
Mean (SD) µg/m^3^	9.4 (2.4)	8.8 (2.1)
**Lung cancer histological subtype (cases only)**		
Adenocarcinoma	294 (32.1)	
Squamous cell carcinoma	228 (24.9)	
Small cell carcinoma	110 (12)	
Other/not specified malignant neoplasms of the lung	285 (31.1)	

Note: Cases were drawn from the Northern Ireland Cancer Registry (NICR), and controls were selected from the NICOLA (Northern Ireland Cohort for the Longitudinal Study of Ageing) study. Individuals aged 85 years and above were collapsed into a single age category ( ≥ 85 years).

### Ambient PM_2.5_ and lung cancer risk

**Fig. 2:**
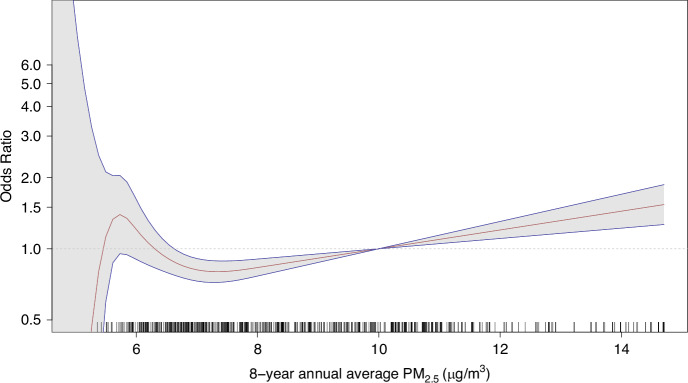
Association between lung cancer risk and continuous residential exposure to particulate matter (PM_2.5_), modelled using a natural cubic spline with five degrees of freedom. The red line represents the estimated odds ratio (OR), the blue lines indicate the corresponding 95% confidence intervals. The model was fully adjusted for age, sex, smoking status, and deprivation quintile. A PM_2.5_ concentration of 10 µg/m³ was used as the reference value. To enhance model stability and clarity of interpretation, six participants with residential PM_2.5_ exposure levels below 5 µg/m³ were excluded. The black tick marks along the x-axis depict the distribution of participants’ annual average PM_2.5_ exposure at their residential addresses.

**Fig. 3:**
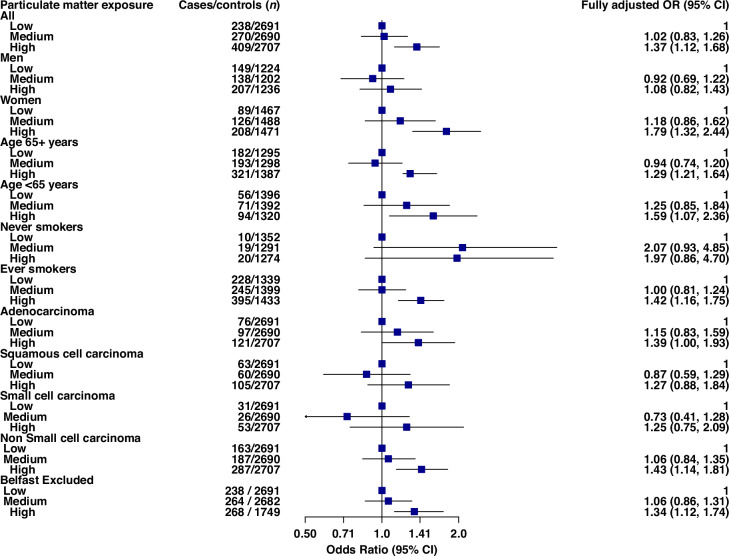
Forest plot displaying the association between particulate matter (PM_2.5_) exposure and lung cancer risk. The analysis includes odds ratios (OR) with 95% confidence intervals (CI) for the main model, as well as stratified analyses by sex, age, smoking status (ever vs. never smokers), and histological subtypes of lung cancer (adenocarcinoma, squamous cell carcinoma, small cell carcinoma large cell carcinoma). Additionally, results are presented after excluding data from Belfast. Cases and controls are reported alongside the fully adjusted ORs.

Odds ratios and 95% CIs for the association of PM_2.5_ and lung cancer are presented in Table 2. Crude ORs (*Model 1*—unadjusted) showed increasing lung cancer risk with increasing PM_2.5_. Though non-significant for ‘medium’ exposure (OR: 1.11; 95% CI: 0.92, 1.33), a significant excess risk was found for ‘high’ exposure (OR: 1.73; 1.47, 2.05). Odds ratios attenuated following adjustment for additional variables but remained significant for ‘high’ exposure in *Model 3* (OR: 1.37; 95% CI: 1.12, 1.68). Findings from the individually matched analysis on these categorical exposures were highly consistent (Fig. S2). For continuous exposure, a significant OR of 1.06 (95% CI: 1.03, 1.10) was found per unit increase in continuous PM_2.5_. The relationship between continuous PM_2.5_ and lung cancer risk is shown in the natural cubic spline plotted in Fig. 2. The PM_2.5_-lung cancer association was stronger among females, for whom a fully adjusted OR of 1.79 (95% CI: 1.32, 2.44) was calculated for ‘high’ exposure. A 12% lung cancer risk increase was found per unit increase in continuous PM_2.5_ in women (*Model 3* OR: 1.12; 95% CI: 1.06, 1.18). The effect of ‘high’ exposure was non-significant for males (*Model 3* OR: 1.08; 95%: 0.82, 1.43) with a significant interaction (*p* = 0.03) found for sex. Results of further stratified analyses are in Fig. 3 demonstrating broadly consistent fully adjusted ORs by age, smoking, histology, and geographic area excluding Belfast, though non-significant for several strata given reduced power. No significant interactions were found for the strata investigated in terms of continuous PM_2.5_ exposure.

**Table 2:** Odds ratios (OR) and 95% confidence intervals (CI) for the association of PM_2.5_ and lung cancer risk in Northern Ireland, for both sexes combined, and males and females separately.

Exposure variable	Category	Number of cases/ controls	Model 1 (crude) OR (95% CI)	Model 2 (minimally adjusted)^a^ OR (95% CI)	Model 3 (fully adjusted)^b^ OR (95% CI)
			*All participants*		
PM_2.5_ (tertiles)	Low (<7.42 µg/m^3^)	238/2691	1	1	1
	Medium (7.42–9.62 µg/m^3^)	264/2690	1.11 (0.92, 1.33)	1.11 (0.92, 1.33)	1.02 (0.83, 1.26)
	High (>9.62 µg/m^3^)	415/2707	1.73 (1.47, 2.05)	1.68 (1.41, 1.99)	1.37 (1.12, 1.68)
PM2.5 (continuous)	Per unit increase (µg/m^3^)	917/8088	1.13 (1.09, 1.16)	1.12 (1.09, 1.16)	1.06 (1.03, 1.10)
			*Females*		
PM_2.5_ (tertiles)	Low (<7.42 µg/m^3^)	89/1467	1	1	1
	Medium (7.42–9.62 µg/m^3^)	126/1488	1.40 (1.06, 1.85)	1.93 (1.04, 1.85)	1.18 (0.86, 1.62)
	High (>9.62 µg/m^3^)	208/1471	2.33 (1.81, 3.03)	2.29 (1.76, 2.99)	1.79 (1.32, 2.44)
PM2.5 (continuous)	Per unit increase (µg/m^3^)	423/4426	1.19 (1.14, 1.24)	1.18 (1.13, 1.23)	1.12 (1.06, 1.18)
			*Males*		
PM_2.5_ (tertiles)	Low (<7.42 µg/m^3^)	149/1224	1	1	1
	Medium (7.42–9.62 µg/m^3^)	138/1202	0.94 (0.74, 1.20)	0.93 (0.72, 1.91)	0.92 (0.69, 1.22)
	High (>9.62 µg/m^3^)	207/1236	1.38 (1.10, 1.72)	1.30 (1.03, 1.64)	1.08 (0.82, 1.43)
PM2.5 (continuous)	Per unit increase (µg/m^3^)	494/3662	1.08 (1.03, 1.12)	1.07 (1.03, 1.12)	1.02 (0.96, 1.07)

^a^Adjusted for age and sex.

^b^Adjusted for age, sex, smoking status, and deprivation.

### Population attributable fractions

**Fig. 4:**
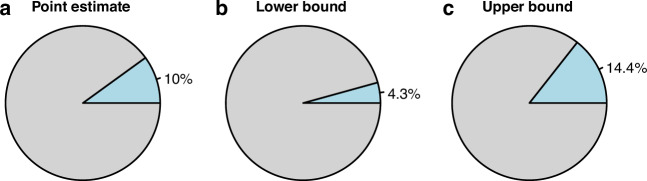
Population attributable fractions (PAF) of avoidable lung cancer cases for both sexes if PM_2.5_ was reduced to <10 µg/m³. Data are presented for (**a**) point estimate; (**b**) lower bound; and (**c**) upper bound.

Population attributable fractions (PAFs) of lung cancer associated with an ambient PM_2.5_ concentration >10 µg/m^3^—the former WHO AQG, for which adherence to is still lacking—were based on the point estimate and lower and upper confidence bounds of the fully adjusted OR for binary exposure above and below this value—1.33 (95% CI: 1.12, 1.56). Pie charts illustrating this range of PAFs are in Fig. 4, and the percentage of lung cancer avoidable if ambient PM_2.5_ concentrations were below this threshold were 10% overall, ranging from 4.3 to 14.4%. Given the nationally representative nature of this study and based on the reasonable assumption that population exposure to ambient PM_2.5_ has remained stable to the present day, we project that this equates to 137 annual cases of preventable lung cancer in NI based on 2022 official statistics [3]—between 56 and 197, which could be avoided if cleaner air policies were implemented.

## Discussion

This study represents the largest population-based cancer case-control study conducted in NI to-date, reporting a positive association between estimated PM_2.5_ exposure and lung cancer risk based on data from 917 cases and 8088 controls. Overall, participants whose residential postcodes were in areas with an 8-year average mapped PM_2.5_ estimate in the highest tertile had a 37% increased lung cancer risk compared to those in the lowest tertile. The effect was clearly detected in women—a 79% increased lung cancer risk for the same comparators—and unclear in men. If we assume the results are unbiased and causal, we estimated that 10% of lung cancers could be avoided if average annual PM_2.5_ concentrations were below 10 µg/m^3^—amounting to a projected air pollution-attributable lung cancer burden of 137 cases annually in NI based on present day statistics, which could be as low as 56 and as high as 197.

While the causal link between ambient air pollution and lung cancer risk is well established [26], our study provides the first direct objective estimates based on long-term ambient air pollution monitoring data and a nationally representative series of cases and controls from NI. Our PAF estimates are similar to those of a less direct nature, including a study estimating the fraction of cancer attributable to modifiable risk factors in the UK in 2015 [4] which reported 5.6% of lung cancer attributable to air pollution in NI, compared to a UK-wide estimate of 7.8%. In their study, Brown et al. employed a relative risk of 1.09 per unit increase in PM_2.5_ and a mean annual concentration of 7.78 µg/m^3^ and 6.9 µg/m^3^ for the UK and NI, respectively to arrive at their estimates.

A notable strength of our study over previous calculations was the fine spatial resolution of the PM_2.5_ maps used for exposure assessment – 1 km^2^, which allowed for a more accurate residence-based exposure characterisation for individuals. Other studies have shown that PAF estimates are sensitive to spatial scale, including a study in France [27] that estimated an air pollution-lung cancer PAF of 3.6% using a 2 km^2^ resolution model. When a nationwide median PM_2.5_ exposure was used, the PAF was underestimated by 72%. Using a lower PM_2.5_ reference level (4.9 μg/m³) increased the PAF to 7.6%, and additional sensitivity analyses resulted in higher values, highlighting the value of high-resolution air pollution data in disease burden assessments.

Geospatial maps are one of the most readily available sources of air pollution data and numerous epidemiological investigations have employed them for particulate matter exposure assessment [28–30]. Our study is consistent with previous geospatial designs conducted at comparable PM_2.5_ ranges in finding an increased lung cancer risk in a population with a relatively ‘low’ (i.e., <15 μg/m³) mean particulate exposure. For instance, the ELAPSE study - a pooled analysis of seven European countries [11]—found a 13% increase in lung cancer risk per 5 μg/m³ of PM_2.5_ concentration, emphasising the important role of our study in further highlighting the substantial burden of lung cancer attributable to ambient air pollution in ‘cleaner’ environments, with associated legislative implications.

In our analysis, adenocarcinoma accounted for 32% of lung cancer cases and represented a greater proportion of female than male cases (37.6% vs. 27.3%, respectively). Notably, higher exposure to PM_2.5_ was more strongly associated with adenocarcinoma than other subtypes, a pattern consistent with findings from European studies such as ELAPSE, ESCAPE, and a meta-analysis by Hamra et al. [11, 31, 32]. These results align with recent global analyses, which report that adenocarcinoma has become the dominant lung cancer subtype worldwide, particularly among women. A 2025 study estimated that in 2022, adenocarcinoma accounted for 59.7% of lung cancers in females and 45.6% in males, with East Asia bearing the highest burden [7]. Moreover, this study provided the first comprehensive quantification of adenocarcinoma cases attributable to ambient PM pollution, estimating that 114,486 cases among males and 80,378 among females could be linked to air pollution exposure. Our finding of a higher air pollution-attributable burden among females in NI is consistent with these emerging statistics, and with some other epidemiological studies [33, 34], but these findings cannot be sufficiently explained within the present design. Possible hypotheses for the stronger signal among females include residual confounding from smoking among men given the crude smoking adjustment used, a higher susceptibility of women to the carcinogenicity of air pollution due to endogenous oestrogen exposure, or smaller airway architecture, and females spending more time at residential locations than males resulting in higher associations with postcode linked PM_2.5._

Our study was limited by some notable weaknesses. While our study focused on PM_2.5_—an established carcinogen in its own right—it was not possible to isolate its effects from those of other co-occurring air pollutants, such as PM_10_, nitrogen dioxide (NO₂), heavy metals, and volatile organic compounds (VOCs), all of which are strongly spatially correlated with PM_2.5_. However, because these pollutants often share common emission sources, policy measures targeting any one of them are likely to yield co-benefits across the broader pollution mixture. Secondly, a lack of residential history for our study participants, which was not available for cases, meant that residential postcode linkage was assumed to be a reliable estimator of long-term exposure. As with all studies using postcode geography, the exposure assessment is ecological in this instance and does not assess exposures away from residence. Notwithstanding this, we note that data from the Northern Irish Longitudinal Study (NILS) have demonstrated that the Northern Irish population experienced a decline in internal migration during the exposure window covered by this study, and that the rate of migration exceeding 10 km distances was below 2% annually [35], suggesting that postcode at diagnosis/recruitment is a reliable indicator of long-term residence for most participants. The limited availability of covariates for lung cancer cases prevented adjustment for factors such as workplace exposures and second-hand smoke which may vary by urban/rural locations and hence confound PM_2.5_ associations, though we expect the contributions of these exposures to be minimal compared to the potential confounding effect of smoking and deprivation, both of which were adjusted for. The adjustment for smoking was crude however, thus residual confounding may be present. Selection bias may also have influenced the results. As cancer registration is complete, but participation rates in NICOLA are not, any selection bias would certainly be differential. E.g. higher participation in NICOLA by women in low PM_2.5_ areas could positively bias the association in women. Nevertheless, comparable PM_2.5_ distributions shared between NICOLA participants and the background NI population are reassuring. We also note that although data employed in this study were collected some ten years prior to analysis, they have relevance to the present day given the enduring nature of the burden of both lung cancer and air pollution exposure locally and worldwide.

Finally, what may instinctively seem a limitation of our study, may be its greatest strength. Our study used two independently collected secondary datasets—lung cancer registrations, and controls from a separate cohort. While it is theoretically preferable to collect data from cases and controls within the same framework, it was possible to harmonise response formats for variables analysed. The synchronous timing of the 2014 NICR lung audit coinciding with the main recruitment wave of NICOLA (2013-2015) meant that cases and controls were recruited within a time period similar to if they were part of a combined study. Furthermore, the nationally representative nature of both data resources makes our PAF estimates widely applicable, a quality rarely achieved by case-control studies. The successful use of these data in this manner paves the way for further investigations into other cancer types and exposures, enabling cost-effective studies on cancer aetiology in the Northern Irish context and acting as an exemplar for other national and international studies. This approach maximizes existing resources and also justifies collecting additional exposure information during cancer registration.

In conclusion, our findings provide consistent compelling evidence of the burden of lung cancer attributable to ambient particulate matter, with broad implications for populations worldwide. They underscore the urgent need for decarbonisation policies, which would mitigate anthropogenic climate change and reduce the incidence of lung cancer and other air pollution-related conditions. Future research should explore whether lung cancer risk models used in population screening could be improved by incorporating geospatial metrics, such as air pollution monitoring data.

## Supplementary information

Supplementary materials

## Data Availability

This study was based on pre-existed routinely collected data of cases and controls from **(NICR)** and (**NICOLA)** accordingly. We gained approval to get a secured access for both datasets. Whereas PM_2.5_ exposure data were publicly accessible from DEFRA at Background Mapping data for local authorities—Defra, UK. Specific ethical approval and consents were not required for this project as all information were anonymous. The datasets generated and/or analysed during the current study are not publicly available due the limits of the ethical approval granted to the **NICR**, to share patient level data. Anonymised, non-patient level data can be made available from the corresponding author on reasonable request.
