# Supplementary material for: Lung cancer burden attributable to ambient particulate matter: a nationally representative population-based case-control study
Source: Br J Cancer. 2025 Oct 6;133(12):1872–9. doi: 10.1038/s41416-025-03207-x (PMC12689774; doi:10.1038/s41416-025-03207-x)
Supplement: Supplementary file 1 — Supplementary materials [file 41416_2025_3207_MOESM1_ESM.pdf]

Supplementary materials

Figure S1: Flow diagram showing the number of exclusions resulting in the final analytical dataset.

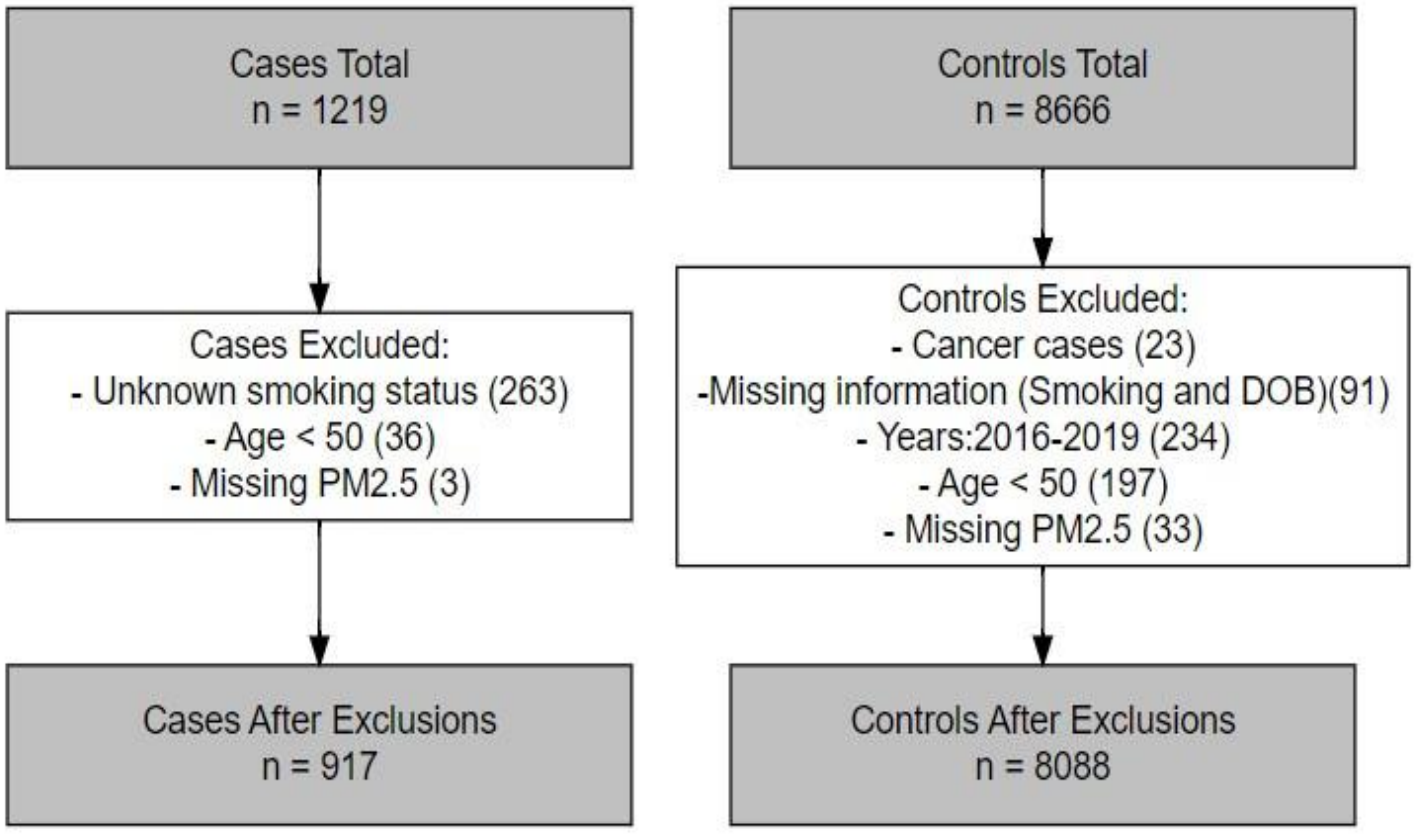

Figure S2: Forest plot showing the association between particulate matter (PM) exposure and the risk of lung cancer in an individually matched analysis by age and sex. This plot presents the ORs and 95% CIs for the association between PM exposure and lung cancer risk, assessed across crude, minimal adjustment, and fully adjusted models.

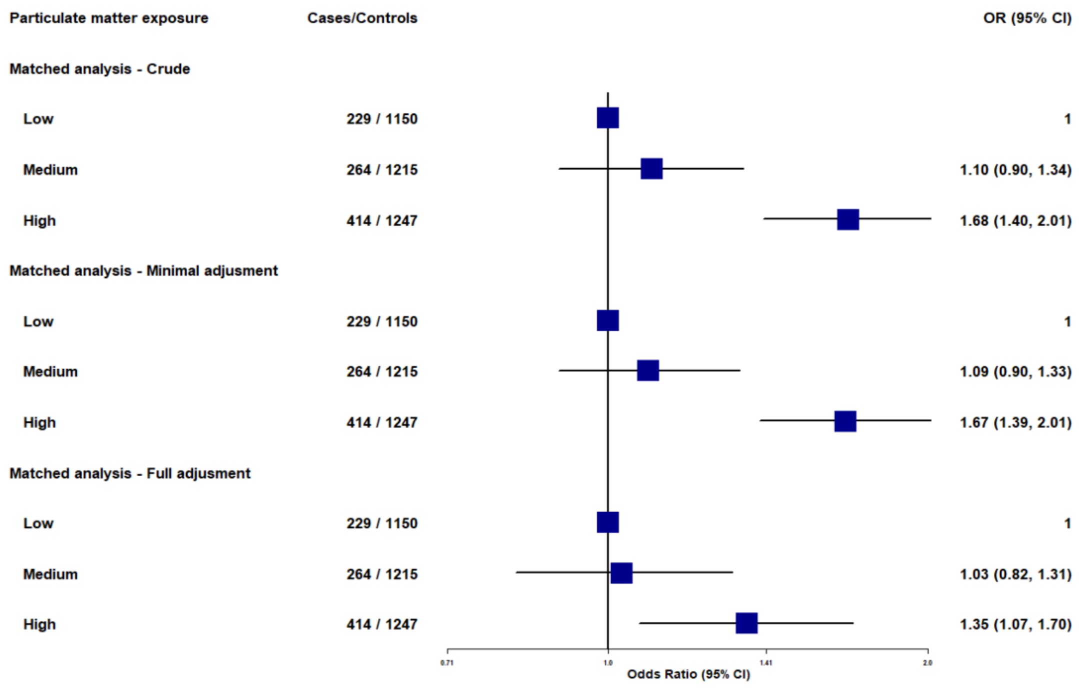

Table S1: To examine the possibility of bias in the context of $PM_{2.5}$ exposure, the representativeness of NICOLA controls used in the present study was assessed by comparing their $PM_{2.5}$ exposure distribution, and mean value, to those of the background Northern Irish population. This was done by expanding the region's Central Postcode Directory by the number of occupants at each residential postcode and joining postcodes to background $PM_{2.5}$ maps in the same manner described for cases and controls (see *Methods - $PM_{2.5}$ exposure characterisation*). This exercise identified no major bias in $PM_{2.5}$ among the sample of controls relative to the population background, supporting their representative. Controls and the background population shared similar means of 8-year average annual $PM_{2.5}$ – 8.8 and 8.9 µg/m^3, respectively, Controls and the background population shared similar means of 8-year average annual $PM_{2.5}$ – 8.8 and 8.9 µg/m^3, respectively, compared to 9.4 among lung cancer cases. Furthermore, the crude odds ratio calculated for 'high' compared to 'low' $PM_{2.5}$ exposure was higher when lung cancer cases were compared to the background population (1.99) instead of NICOLA controls (1.73).

**Sample group**	**8-year average annual $PM_{2.5}$ distribution, *n* (%)**			**Mean 8-year average annual $PM_{2.5}$ (µg/m^3)**
	**Low (<7.42 µg/m^3)**	**Medium (7.42-9.62 µg/m^3)**	**High (>9.62 µg/m^3)**	
**Cases (*n*=917)**	238 (26.0%)	264 (28.9)	415 (45.3)	9.4
**Controls (*n*=8,088)**	2,691 (33.3 %)	2,690 (33.3%)	2,707 (33.5%)	8.8
**NI background (*n*=1,587,229)**	494,956 (31.2%)	557,948 (35.2%)	534,325 (33.7%)	8.9

Table S2: $PM_{2.5}$ tertiles in controls, overall and by potential confounding factors.

Category	8-year average annual $PM_{2.5}$, n (%)			*p*-value (Chi-square test)
	Low (<7.42 µg/m³)	Medium (7.42-9.62 µg/m³)	High (>9.62 µg/m³)	
All	2691 (33.5)	2690 (33.6)	2707 (33.9)	
**Age (years) at interview**				0.02
50-54	419 (15.6)	455 (16.9)	426 (15.7)	
55-59	500 (18.6)	472 (17.5)	474 (17.5)	
60-64	477 (17.7)	465 (17.3)	420 (15.5)	
65-69	478 (17.8)	426 (15.8)	468 (17.3)	
70-74	368 (13.7)	334 (12.4)	360 (13.3)	
75-79	230 (8.5)	248 (9.2)	251 (9.3)	
80-84	129 (4.8)	181 (6.7)	178 (6.6)	
85-89	63 (2.3)	82 (3.0)	95 (3.5)	
90-94	25 (0.9)	23 (0.9)	31 (1.1)	
95-99	2 (0.1)	4 (0.1)	4 (0.1)	
**Sex**				0.75
Male	1224 (45.5)	1202 (44.7)	1236 (45.7)	
Female	1467 (54.5)	1488 (55.3)	1471 (54.3)	
**Smoking status**				<0.001
Current	386 (14.3)	442 (16.4)	522 (19.2)	
Ex-smoker	953 (35.4)	957 (35.6)	911 (33.7)	
Never	1352 (50.2)	1291 (48.0)	1274 (47.1)	
**Deprivation class**				<0.001
Quintile 1 (Least deprived)	136 (5.1)	790 (29.4)	847 (31.3)	
Quintile 2	660 (24.5)	687 (25.5)	402 (14.6)	
Quintile 3	1101 (40.9)	373 (13.9)	332 (12.3)	
Quintile 4	684 (25.4)	484 (18.0)	401 (14.9)	
Quintile 5 (Most deprived)	110 (4.1)	356 (13.2)	725 (26.8)	
